# Sentimental Analysis of COVID-19 Related Messages in Social Networks by Involving an N-Gram Stacked Autoencoder Integrated in an Ensemble Learning Scheme

**DOI:** 10.3390/s21227582

**Published:** 2021-11-15

**Authors:** Venkatachalam Kandasamy, Pavel Trojovský, Fadi Al Machot, Kyandoghere Kyamakya, Nebojsa Bacanin, Sameh Askar, Mohamed Abouhawwash

**Affiliations:** 1 Department of Applied Cybernetics, Faculty of Science, University of Hradec Králové, 50003 Hradec Králové, Czech Republic; venkatachalam.k@ieee.org; 2 Department of Mathematics, Faculty of Science, University of Hradec Králové, 50003 Hradec Králové, Czech Republic; pavel.trojovsky@uhk.cz; 3 Faculty of Science and Technology, Norwegian University for Life Science (NMBU), 1430 Ås, Norway; fadi.al.machot@nmbu.no; 4 Institute for Smart Systems Technologies, Faculty of Technical Sciences, Universitaet Klagenfurt, A9020 Klagenfurt, Austria; kyandoghere.kyamakya@aau.at; 5 Faculty of Informatics and Computing, Singidunum University, Danijelova 32, 11000 Belgrade, Serbia; nbacanin@singidunum.ac.rs; 6 Department of Statistics and Operations Research, College of Science, King Saud University, Riyadh 11451, Saudi Arabia; 7 Department of Computational Mathematics, Science, and Engineering (CMSE), College of Engineering, Michigan State University, East Lansing, MI 48824, USA; 8 Department of Mathematics, Faculty of Science, Mansoura University, Mansoura 35516, Egypt

**Keywords:** COVID-19, data prediction, N-gram feature extraction, ensemble machine learning, twitter data

## Abstract

The current population worldwide extensively uses social media to share thoughts, societal issues, and personal concerns. Social media can be viewed as an intelligent platform that can be augmented with a capability to analyze and predict various issues such as business needs, environmental needs, election trends (polls), governmental needs, etc. This has motivated us to initiate a comprehensive search of the COVID-19 pandemic-related views and opinions amongst the population on Twitter. The basic training data have been collected from Twitter posts. On this basis, we have developed research involving ensemble deep learning techniques to reach a better prediction of the future evolutions of views in Twitter when compared to previous works that do the same. First, feature extraction is performed through an N-gram stacked autoencoder supervised learning algorithm. The extracted features are then involved in a classification and prediction involving an ensemble fusion scheme of selected machine learning techniques such as decision tree (DT), support vector machine (SVM), random forest (RF), and K-nearest neighbour (KNN). all individual results are combined/fused for a better prediction by using both mean and mode techniques. Our proposed scheme of an N-gram stacked encoder integrated in an ensemble machine learning scheme outperforms all the other existing competing techniques such unigram autoencoder, bigram autoencoder, etc. Our experimental results have been obtained from a comprehensive evaluation involving a dataset extracted from open-source data available from Twitter that were filtered by using the keywords “covid”, “covid19”, “coronavirus”, “covid-19”, “sarscov2”, and “covid_19”.

## 1. Introduction

Gathering of people opinion and analyzing data in social media has interesting facts due to its real time interactive in nature. Due to that reason, current research work has relied on social media networks as well as sentiment analysis in order to tracking people’s behaviour and opinions about current scenario. Information of corona-virus was distributed across social web sites. Researchers had recently implemented sentiment analysis to classify attitudes of people from tweets based on healthcare corona-virus pandemic sentiment analysis. Social media networks are presenting various views, emotion and opinion of all users. These sentiment analyses will produce remarkable findings [1,2]. This isolation of people pushes them to explore other activities and to more sharing the news across social media applications like Facebook, twitter and WhatsApp. Social media play a significant part by connecting people virtually during this pandemic situation and for communications regarding the spread of diseases. The heavy usage of social media brings all views on Covid-19 into some centralized server as a message. There are various types of messages like positives, negatives and medical devices in the highest rates [3,4]. The loss of human life in covid 19 creates a massive challenge to the human survival as many are without proper work, education, and sufficient food systems. More countries both developed and underdeveloped ones face economic crisis and social problems in their societies. The number of people affected by poverty, under nourishment and unhealthy food habits has significantly increased to 132 million by 2020 [3]. Important news like mortality, virus spread in countries, vaccinations, medicines, monetary helps, etc. are presented amongst the various social media with specific differences which does express another reason for the virus spread [4].

Daily, the world faces different events such that new situations arise based on diseases spread. A significant part of the huge information we share in social media is regarding our way of life, relationships and various comments and announcements related to the virus and its spreads. Some days are particularly prominent, were people fear that this dangerous virus is not stopping to spread [5]. Broad issues and challenges based on control measures by governments and NGO’s are highly broadcasted in the media [6,7]. At present, most health communities are highly affected by the COVID crisis [8]. Our technological world, with its various advancements of adaptability, free travels between nations, and events coordination, makes of covid-19 crucial and sensitive situation in society [8]. The related financial progress/developments in various countries and their economic connectivity are highly featured in social media [9].

On one side, the virus outbreak seems critical, on the other side, fake news, wrong guidance, and criticism are going around social media. This misguidance results in huge problems for the government’s health sectors for them providing the right information to the people. Both health ministries and the broad masses of people face high tension due to the huge misinformation broadcasted in the in the social media [10]. A sentimental analysis of the social media regarding COVID-19 based on five different classification criteria can be done with the positive and negatives in the words [11,12,13,14,15]. In this research, large datasets with 312 million tweets of COVID-19 in English language and sentiment scores are considered for a comprehensive sentimental analysis. This analysis is performed using a so-called N-gram autoencoder integrated within an ensemble machine learning scheme. This “ensemble” scheme integrating an N-gram autoencoder is a new deep learning technique for textual data analysis.

In the research presented in this paper, we find comprehensive answer for the following research questions:

- Q1: What are the most popularly used keywords in social tweets on covid-19?
- Q2: What are the reasons that some keywords create/induce negative thoughts in the population w.r.t. the public health system?
- Q3: How far can ensemble learning improve the efficiency of analyzing emotions through text analysis of the messages in social media?
- Q4: How far does our novel proposed model N-gram auto-encoder model integrated in an ensemble learning scheme outperforms, after a comprehensive benchmarking selection of the best competing previous/existing relevant models?
- Q5: How far is the comprehensive analysis conducted in this research helpful and of some significance to society in general?

This paper is globally arranged as follows. A comprehensive critical literature review is presented in Section 2. Then, relevant materials and methods, including our novel suggest scheme, are presented in Section 3. Furthers, performance evaluation results and a comprehensive benchmarking are discussed in Section 4. And finally, a series of concluding remarks summarizing the quintessence of this paper, especially the comprehensive responses to the core research questions to be answered by this paper are presented and explained in Section 5.

## 2. Related Work

In the social network of Twitter, population can share their view, thoughts and posts about the current scenario in the trending society, such as the corona virus. In the tweet posts COVID-19 has become a trending keyword-based tweet which contains more information about corona virus [16,17]. For the given tweet document sentiment, analysis plays a vital task in classifying the polarity score which indicates to express the people opinion like positive, negative or neutral. Beyond that sentiment analysis people can share their emotions like anticipation, anger, fear, sadness, joy, trust and disgust [18,19]. From this tweet information public health authorities can monitor, behaviors, surveillance of health information and it reduce the pandemic’s impact. Similarly understanding people’s needs, this data can help health care workers in monitoring and health data surveillance, behaviors, and planning intervention to decrease pandemic impact. Knowing population needs with their discrimination and fulfillment by taking preventative steps, to overcome the situation of COVID-19 [20].

Major global issue is increase of false news in social media which affects the health department, society life through social media. Fake news always disseminates novel ideas and real measures needed to be taken for reducing the pandemic [21]. Fake news psychologically affects people mind and creates unnecessary fear regarding COVID 19 pandemic. This highly effects government measures and health workers to make people highly positive in fighting pandemic [22]. Misinformation circulates in social media creates panic among corona patients which create big work on public authority to advice citizens about genuine steps and fake circulating stories [23]. Research paper [24,25,26] presents regarding behavior of people due to social media news about the pandemic. Pandemic related individual assessment [27] for overcoming rumors must be deployed using technologies.

Chakraborty K et al., 2020 describes positive tweets on covid-19 creates good sentiments but some Users are wontedly engaged in spreading negative news for affecting society and politics [28,29]. Here the word frequency calculation used in measuring the words in the social tweets. Machine learning approaches for sentimental analysis [30] perform automatic detection and look for good frameworks which can predict false instances in social media. Covid 19 news face big effects when it is misused for political ideology which can poison public health [31]. Huynh 2020 presents enormous rumor related data which circulated across globe regarding COVID-19. Analysis and differentiation of false information from true information with high accuracy is a big question. This validation process can help people and health care workers from unwanted pressures. In this research article we implemented deep machine-learning models, to validate the news high accuracy.

## 3. Proposed Deep Learning-Based Pandemic Prediction

**Figure 1:**
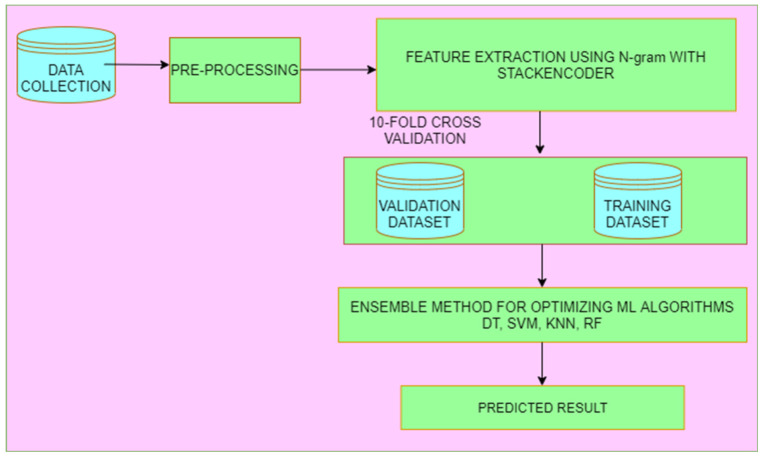
Proposed architecture for N-Gram Stacked Auto-Encoder.

In this research, pandemic prediction using deep learning architecture in social media is presented by following techniques such as corona prediction, analysis and detection. For implementation details, we used the tweets’ dataset was collected and filtered by #COVID-19, #Coronavirus and #COVID’19 hashtags. We explored the deep learning concept in real-time system for predicting COVID-19, and it has been developed into two phases. First, we perform Sentiment analysis with Latent semantic analysis pre-diction in offline mode. Secondly, we Exhibit a model in the online mode. The overall framework is given in Figure 1.

### 3.1. Sentiment Analysis with Latent Semantic Analysis Prediction in Offline Mode

The sentiment analysis purpose is to automatically pick out whether or not a given piece of textual content to articulate opinions like positive or negative on topic of interest. Latent semantic analysis (LSA) is used to retrieve the useful data from the text. Offline sentiment and semantic model for analysis have been designed to examine the machine learning techniques to identify the optimal solutions. The AI model which has been used are the K nearest neighbour (KNN), random forest (RF), decision tree (DT) and support vector machine (SVM). In this proposed work, we implemented ensemble learning method, which combine DT, SVM, RF, KNN predictive models and then combine all predictions by using statistics, such as the mode or mean to produce improved results. These techniques have been trained and tested using tweets dataset of the corona-virus.

#### 3.1.1. Data Collection

In this work Twitter data was collected from open-source available from IEEE website [32]. This freely available dataset contained global tweets and filtered by using keywords “coronavirus”, _covid”, “-covid-19”, “sarscov2”, “#covid19”, “#covid_19”, “2019-ncov”, “#2019ncov”, “sarscov2”, “#covid”, “sarscov2”, “sars cov2”, etc. Tweet IDs were available only from 20 March 2020 [28]. From the IEEE website, capturing the information about tweet extract the tweet ID is called tweet objects. Tweet object contains created time of tweet, tweet text, tweet ID, status of retweeted, location etc. are in JSO format [33]. Using DocNow hydrator tool Tweet ID were hydrated in the format of JSON and CSV [34]. The hydrated covid_tweets were downloaded from 20 March 2020 to 20 April 2020, as CSV file format. For measuring the polarity score in the sentiment analysis Valence Aware Dictionary and sentiment Reasoner (VADER) act as lexicon and tool for rule-based sentiment analysis are used to detect positive, negative and neutral comments [35] as shown in Table 1. The scoring can be calculated as compound scores ϱ1 is evaluated by adding all words valence score in the lexicon and normalized the value between $Y_{max}$ and $Y_{min}$. Here, $Y_{max}=1$ denotes most extreme positive and $Y_{min}=-1$ denotes most extreme negative. For classifying the sentence positive, negative, and neutral by using threshold value. 

For positive sentiment Score: ϱ1≥0.05.

For Negative Sentiment Score: ϱ1≤−0.05.

For Neutral sentiment Score: ϱ1>−0.05  and ϱ1<−0.05.

#### 3.1.2. Data Preprocessing

**Figure 2:**
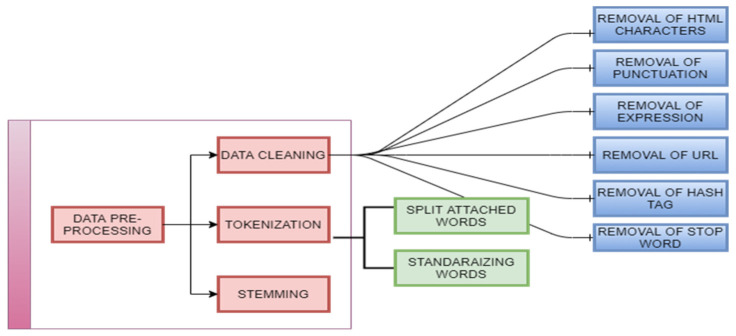
Framework of data preprocessing.

Pre-processing data are a vital role in the social media network concept analysis system. That is in sentiment analysis and latent semantic analysis of streaming data of Twitter. The text data available via Twitter are highly unstructured and noisy in nature. To achieve the best result, data preprocessing is required. The steps of data preprocessing are given in Figure 2.

1. Data Cleaning: in this phase, unwanted contents are removed using the following steps:
  - Removal of HTML characters: the web data usually contain a lot of HTML entities such as <>&, which are embedded in the original data. By using the HTML parser of Python, we can convert these entities into standard HTML tags. For example, < is converted to “<”, & is converted to “&”, and so on.
  - Removal of Punctuation: All the punctuation marks consistent with the priorities must be dealt with. For example, “.”, “,”, and ”?” are essential punctuations that need to be retained, while others need to be removed.
  - Removal of Expressions: the text data might also contain human expressions such as laughing, crying, and some emojis. These expressions are generally not applicable to the content of the text and as a result need to be removed.
  - Removal of URL: In this step, we remove URLs and hyperlinks in text data such as comments and reviews, which are irrelevant to the process.
  - Removal of Hash Tags: to access the content of twitter statement by using # symbolic notation. This hashtag is act as an index or keywords for accessing Twitter content. Example #COVID-19- and #coroanvirus etc. These hash tags are removed.
  - Removal of Stop Word: in the text analysis, stopping words are not applicable. We have to remove or filtered such stop words like conjunctions, prepositions, articles.
2. Tokenization: it breaks up the longer strings or sentences into smaller pieces or tokens. It involves two steps.
  - Split Attached Words: the first step involves generating text data in the social network in an informal structure. Most of the tweets contain attached words such as “its pandemic”, “fully lockdown day” and so on. These entities can be split into normal forms.
  - Standardizing Words: the textual data are not in proper format such as “misssss u”, “loveeeee u.” We must break these sentences into their proper format.
3. Stemming: it is process of converting the words into their original form. That is decreasing the number of words from root to word type of text. For example, the words “Jumping,” “jumped,” will be cut-off to the word “jump.”

#### 3.1.3. Feature Extraction

In the analysis of textual data feature extraction is challenging one. Text feature extraction that extracts text information from the large number of text processing to represent a text message [35]. some effective ways are identified for reducing the feature space dimensions and this process known as feature extraction [36]. During feature extraction, we delete uncorrelated features [37]. In this proposed work, we introduce a novel deep learning methodology using stacked encoders to identify sentiment and latent sematic analysis at the word level. The proposed new model is distributing the word vector representation by “n” gram as input and the resulted continuous word vectors are combined with stacked auto encoder for fine-tuning of word embeddings.

#### 3.1.4. N Gram

N-Gram is a supervised machine learning algorithms for feature extraction of text. In given textual information, this N is considered as single bit of information or tokens. 

If N=1 for unigram; N=2 for bigram and N=3 for trigram and so on. 

Following steps are followed in our process:

1. Gathering of twitter API text data based on #tag related to COVID’19.
2. Using the dynamic analysis extract the features from the executable files.
3. Create N-grams for N = 1, 2, 3
4. Reducing feature space by create a vector of tokens in randomised order.
5. Extracting the string information from the textual data using N-Gram approach.

#### 3.1.5. Stacked Autoencoder

**Figure 3:**
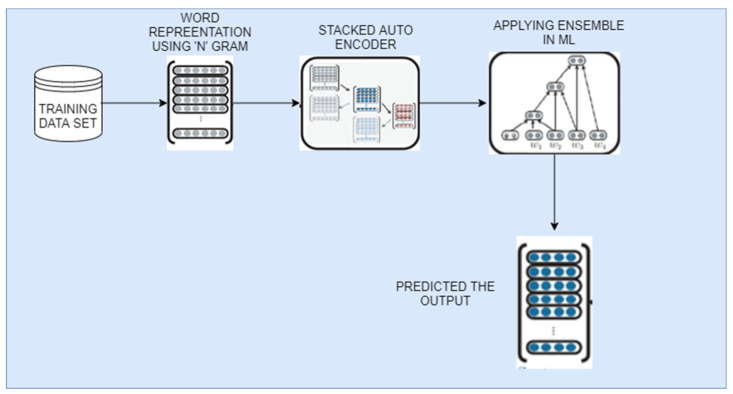
Feature Extraction process Architecture.

1. N-gram string information is distributed and contains frequently used words.
2. Then, the attacked autoencoders “SA” algorithm is used to convert the representation into a reduced vector.
3. The sentiment and latent semantic analysis are used with machine learning algorithms such as decision tree (DT), support vector machine (SVM), random forest (RF), and K-nearest neighbor (KNN).
4. Implementing the ensemble method for the above ML model produces the prompt prediction. Figure 3 shows the framework of feature extraction.

To extract the features of Twitter API streaming text files generated by dynamic analysis, we are developing the following Algorithm 1. The Cd contains the set of textual Twitter data, *W_i_* and *W* are a set containing both words with a COVID-19-related hashtag; thus, we can write:

(1) Cd=W

where $W=\left\{w_{1},w_{2},\ldots w_{n}\right\}$.
**Algorithm 1.** Data: Dataset ’Cd’: contains Twitter API streaming COVID’19**Step 1:** Read Twitter API text data. **Step 2:** Begin. **Step 3:**$\;\mathrm{For}\;\mathrm{each}\;w_{i}\in Cd$ do. **Step 4:** Using dynamic analysis generate behaviour analysis textual information. **Step 5:** In the behaviour analysis textual information extracting the API calls function with argument values and omit the remaining Features. **Step 6:** Creating ‘3-grams’ for API call function with arguments. **Step 7:** Make a sorted table of API n-grams according to the frequency of occurrence. **Step 8:**$\;\mathrm{For}\;\mathrm{each}\;w_{i}\in Cd\;$ do. **Step 9:** For each API n-gram, do feature vector. **Step 10:** Create binary feature vectors for n-grams. **Step 11:** For each binary feature vectors of n-gram, do stack autoencoder **Step 12:** Train the autoencoder with its binary input data and produce the feature vector of n-gram. **Step 13:** The feature vector value of the previous layer is used as the input for the successor layer, and it is repeated until the training process completed. 
$\;\;\;\;\;\;\;\;\;{\left\{s_{n}\right\}}_{n=1}^{N},\;where\;s_{n}\in D^{m\times 1}$    (2) 
$h_{n}$ denotes the hidden encoded vector value calculating from
$s_{n}$. 
$\hat{w_{n}}$ is the vector value of decoder for the output layer. Therefore, the process of encoding is given below: 
$\;\;\;\;\;\;\;\;\;h_{n}=f\left(W_{1}s_{n}+b_{1}\right)$        (3) where *f* is function of encoder,
$w_{1\;}$ is the function weight matrix and $b_{1}$ is the bias vector value. **Step 14**: Processing Decoder $\;\;\;\;\;\;\;\;\;\hat{s_{n}}=g\left(W_{2}h_{n}+b_{2}\right)$        (4) 
where, *g* is the function of decoder *W*_2_ is the weight matrix *b*_2_ is the bias vector value. **Step 15:** After training all hidden layer cost function and weight updating is done using, backpropagation network (BPN) with labelled fine-tuned training set of the predicted output.

#### 3.1.6. Stratified 10-Fold Cross-Validation

The Twitter dataset is classified using 10-fold cross-validation, which consists of 80% training data and 20% test data. The corpus data of Twitter includes the collection of textual data with the #COVID-19 and #coronavirus hashtags. The training data set is used to optimize the process by using search technique based on grid of 10-fold cross-validation (CV) has been exposed to identify the optimized result of prediction by using 3 different types of ML algorithms.

#### 3.1.7. Ensemble Method for Optimization ML Algorithm

An ensemble method combines several machine learning and meta-algorithmic results into predictive model for increasing prediction accuracy. In this work, we are implementing ensemble learning, because of the improvement and robustness of machine learning algorithms like decision tree (DT), support vector machine (SVM), random forest (RF), K-nearest neighbour (KNN). It combines the predictions from DT, SVM, RF and KNN. The prediction result of above ML techniques is then ensemble with bagging method known as max voting for final output prediction. This bagging concept is implemented by

(5) $$f\left(x\right)=\frac{1}{m}\sum_{i=1}^{m}f_{i}\left(x\right).$$

**Algorithm 2.** (Proposed N-gram with Stacked Auto Encoder)**Input:** tweets dataset **Output:** result prediction **Step 1**: Pre-processing the input data set using the Section 3.1.2 (Pre-Processing) **Step 2:** for performing the Feature Extraction the pre-processed input data are then given as input in Algorithm 1. (Feature Extraction) **Step 3:** Machine learning: the selected feature data send as input to classification models such as DT, SVM, RF and KNN. **Step 4:** Ensemble method of Learning: the algorithms prediction results are calculated separately. The maximum result in prediction is considered as final prediction outcome of the proposed method. Max (output (DT), output (SVM), output (RF), output (KNN)) **Step 5:** Final outcome: prediction result is returned.

### 3.2. Exhibiting a Model in Online Mode

The model in online sentiment and latent semantic pipeline component prediction aims for tweet prediction related to coronavirus in real time and implement the model to work in real-time process. To perform real-time processing, it collects streaming tweets and fed it to the Machine Learning model to predicting the sentiment analysis and latent semantic analysis of the coronavirus tweets. For that we are using TensorFlow library in Python. It is based upon feedforward neural net concept. It classifies the coronavirus tweet vector in negative or positive using the following steps.

**Step 1:** The neurons in the input layer have a tweet vector, so every neuron is linked with one word in the lexicon. The weighted sum of each neuron is fed through the ReLU activation function. The rectified linear unit (ReLU) is mathematically defined as:

(6) $$Y=max\left(0,X\right).$$

**Step 2:** There are two neurons are available in the output layer, along with a SoftMax activation function, and it produces the result as either negative or positive.

(7) $$\sigma \left(\vec{z_{i}}\right)=\frac{e_{i}^{z}}{\sum_{j=1}^{k}e_{j}^{z}}$$

**Step 3:** For classification problems such as the sentiment and latent semantic analysis, there are two hidden layers for producing the training result.

For prediction pipeline at online, components are developed using Twitter streaming API along with a message distributed system of Apache Kafka and Apache Spark (big data process).

## 4. Results and Discussion

### 4.1. Dataset Description

The result evaluation of the proposed work for sentiment analysis in real time and latent semantic examination has been developed using Python with Spark’s Mlib to execute machine learning techniques of RF, DT, SVM, and KNN [32]. Twitter data streaming of API is used for data collection from Twitter and Apache Kafka was used for receiving data streaming from the server. This work uses streaming API from 20 March 2020, to 20 April 2020. Twitter streaming data is filtered by using standard keywords like “#covid’19”, “corona”, “coronavirus”, “#corona” and “#coronavirus”. Number of tweets collected per day in average is around 923k. Table 2 shows that overview of filtering tweets by using the sample keywords.

Table 2 shows the process for filtering tweets related to COVID-19. Keywords that are matched with tokenized text of the COVID-19 tweets are filtered. Using unigram, bigram, and trigram, we can filter the tweets.

### 4.2. Sentiment Prediction

The predictions of the COVID-19 tweets by using unigram, bigram, and trigram for the respective time span is shown below. The N-gram model (N = 3) captures sentiments expressed by using some emojis. Table 3 shows samples of sentiment analysis based on COVID-19 emojis shared via tweets.

Table 3 shows that most of the tweets are based on folded hand, index pointing, and backhand-index pointing, through which people shared their opinion and feelings about COVID-19. Some tweets are optimistic, pessimistic, sad, angry, feeling bad, and so on.

### 4.3. Performance Metric Measures

The performance of the system is calculated using the following metrics.

Four standard metrics were applied to evaluate the accuracy, precision, recall, and *F1-score*; here, *TP* is true positive, *TN* is true negative, *FP* is false positive, and *FN* is false negative, as given in the following equations:

(8) $$accuracy=\frac{TP+TN}{TP+TN+FP+FN}$$

(9) $$precision=\frac{TP}{TP+FP}$$

(10) $$True\;Positive\;Rate\;or\;Recall=\frac{TP}{TP+FN}$$

(11) $$False\;Positive\;Rate=\frac{FP}{FP+TN}$$

(12) $$F1-Score=\frac{2*precision*recall}{precision+recall}$$

(13) $$Error\;Rate=\frac{1}{n}\sum_{i=1}^{n}{\left(Y_{i}-{\hat{Y}}_{i}\right)}^{2}.$$

For developing the offline mode of the system and evaluate the analysis to identify the optimal techniques in machine learning for the best performance in real-time prediction of the sentimental analysis and latent semantic polarity. We study the four techniques in ML of RF, DT, SVM, and KNN [32] performance using tweet dataset, from IEEE website which is related to coronavirus and has hashtags are #Coronavirus, #COVID’19, #COVID-19. The four machine learning classifiers were executed using package of Scikit-learn 0.21.3 in Python3.7. for classification. The 10-fold cross-validation is used for tuning the hyper parameter and training the model. By using these four machine learning techniques were used to trained with 80% of data and then tested with the remaining 20% of data. The ensemble-based prediction outcome of these machine learning algorithms are implemented by using the bagging technique called max voting to predict the final outcome.

In this paper, we considered feature extraction using N-Gram-Stacked Autoencoder (value of N = 3) on the Twitter sentiment analysis and latent semantic analysis dataset. Table 4 shows the output for four performance parameters i.e., precision, recall, and f1-score of four classification techniques are the decision tree (DT), support vector machine (SVM), random forest (RF), K-nearest neighbour (KNN) using Unigram staked auto-encoder.

Table 5 shows the output for four performance parameters i.e., precision, recall, and f1-score of four classification techniques are decision tree (DT), support vector machine (SVM), random forest (RF), K-nearest neighbour (KNN) using Bigram stacked autoencoder.

Table 6 shows the output for performance parameters, i.e., precision, recall, and F1 score, of four classification techniques (decision tree (DT), support vector machine (SVM), random forest (RF), and K-nearest neighbor (KNN)) using the trigram stacked autoencoder.

**Figure 4:**
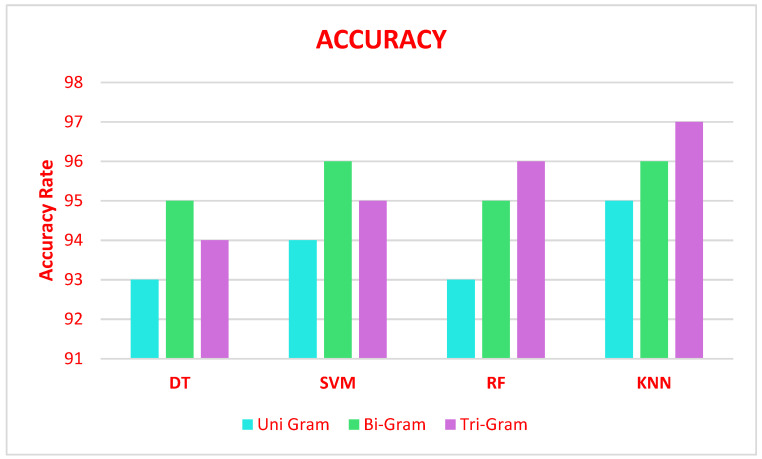
Accuracy of ensemble models (DT, SVM, RF, and KNN) in predicting COVID-19 severity with N-gram stacked autoencoders.

The performance evaluation of various ML algorithms such as DT, SVM, RF, and KNN algorithm on N-gram stacked autoencoder feature extraction obtains the accuracy illustrated in Figure 4.

**Figure 5:**
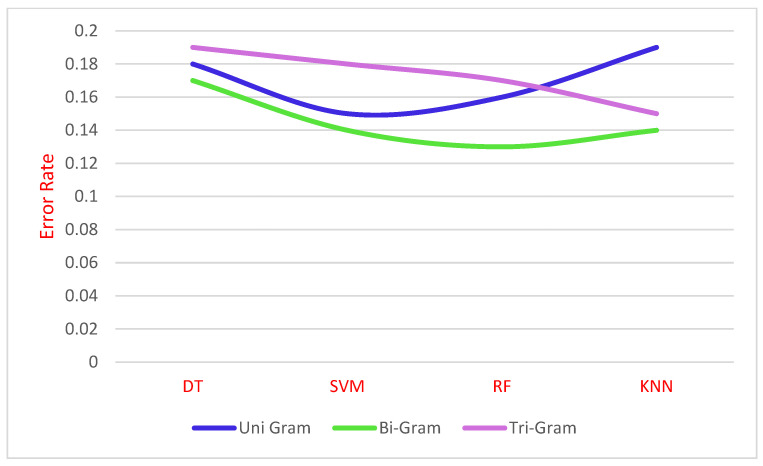
Error rate of the N-gram stack autoencoders.

Figure 4 to explain N-Gram stacked auto-encoder accuracy with various classifiers of DT, SVM, RF and KNN based on Algorithm 2. The result of these classifiers algorithms is then the ensemble using the bagging technique called max voting to predict the final result. Figure 5 shows that error rate of various classifiers with N-Gram stacked auto encoder. This max voting concept produced the validity and reliable model for the sentiment analysis of COVID data set.

**Figure 6:**
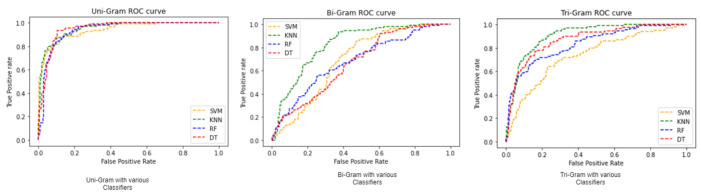
ROC for SVM, KNN, RF, and DT classifiers.

Figure 5 shows the error rate of classifiers DT, SVM, RF, and KNN with N-gram stacked autoencoder. The error rate is calculated by comparing the sending and received words. In this work, prediction is compared with the real opinion of the public. Figure 6 shows that ROC curve of the unigram, bigram, and trigram stacked autoencoders using four classifiers.

For evaluation of performance metric measures, we used true positive, true negative, false positive, and false negative values in order to calculate the accuracy, precision, and recall. In Figure 6, the true positive rate and false positive rate can be seen for various classifiers namely SVM, RF, KNN and DT with N-Gram stacked auto-encoder. These are applied in training dataset; the true positive rate is high which shows that the effectiveness of the classifiers.

### 4.4. Analysis Internal and External Threats in Overall Sentiment

People can share their opinions, feelings, sharing trending topics, current situation in the social media freely. At the same time, it is necessary to detect the potential internal threat and external threats. In this work, we collected all tweets textual information and classified the tweets by the stages of threat based upon the criteria. Sentiment level was calculated for all tweets. Calculate the average sentiment score of tweet and ratio of negative sentiment tweets. Then detect the inside and outside threats based on the concept of information security compliance. Internal threat was categorized into three stages: High, Medium and low. If the negative sentiment was set to -0.2 and ratio of negative tweet was set 40% [37,38,39]. In our data set by using decision tree algorithm which detect threats in inside and outside in an efficient way [40]. Table 7 shows that classification of threat level.

Table 7 describes the threat level in the sentiment analysis on social media. The criteria available in Table 7 are used to detect the malicious internal threats. Table 8 shows that sample of detecting the threats.

Table 8 describes the detecting of threats in three stages based on the values of sentiment score [41,42] and ratio of negativity tweets.

## 5. Conclusions and Future Work

This research article has focused on a comprehensive real-time sentimental data analysis and predictions based on streaming data from Twitter, which are related to the dangerous COVID-19 pandemic. In this work, we are using the most standard classifiers: DT, KNN, RF, and SVM. The proposed N-gram stack autoencoder integrated within an ensemble machine learning scheme has been developed, validated, and benchmarked with competing schemes from the most recent and relevant literature. Hereby, the streaming API of Twitter, apache spark, and kafka were involved. One computes/performs a comprehensive sentimental analysis of those data both in offline and online mode. The matching process is computed/trained offline for related components. Then, in the online mode, the trained components involving the n-gram stacked autoencoder integrated in an ensemble machine learning scheme are used. The results have been evaluated/benchmarked/compared with five machine learning schemes involving various bigram and unigram models. When compared with existing algorithms, our proposed n-gram stacked autoencoder in the ensemble machine learning improves the accuracy and time required. The datasets were collected from streaming API since 20 March 2020; they were extracted by using the hash tags #covid-19 and #coronavirus. In social media, some tweets based on COVID contain information about death analysis or the rate and severity of COVID, which would induce negative thoughts. The performance analysis results outcomes show and validate that our novel proposed work significantly outperform all other competing techniques/schemes while considering accuracy, precision and recall. Our scheme reaches an accuracy of 87.75%, which is 4% to 10% greater than all the other competing related techniques. The comprehensive analysis of this proposed work provides information to society about taking precautions and helps people find reliable information about COVID-19 and connect with one another.

The research findings for Q1 to Q5 state that the most widely used tweets are #Covid 19, #lockdown, #oxygen, #cylinders, #oximeter, #death, #vaccination, #WHO, #stay, #safe, #mask, #sanitizer, #PCR, #test, #recovered, etc. In addition, negative tweets include #death, #No beds, #no oxygen beds, #cannot recover, #more death, #alcohol, #poison, #bad government, #no job, #economy down, etc. The accuracy of classifying negative tweets and COVID tweets is improved using N-gram stack autoencoder compared with the ensemble model techniques with simple N-gram. These negative tweets can alert medical teams and the government to the emotions among the living population. Further knowing the emotions and opinions of the population, the government can generate positive news across social media and make people confident.

Our future works in this research will involve advanced deep learning schemes and various classifiers with the purpose of further improving accuracy (e.g., significantly beyond 90%) in the comprehensive sentimental analysis of social media messages with regard to the COVID-19 epidemic.

## Figures and Tables

**Table 1:** Example for scoring the sentiment polarity by VADER.

Social Tweet	Sentiment
Received result as positive for COVID-19. Home quarantine and stay home for all the brave	Positive
COVID Positive, no mental strength to tackle this situation. Depends on caretaker	Negative
Wearing Face Masks is needed for every human being.	Neutral

**Table 2:** Sample filtering keywords for COVID-19.

Period Interval	Keywords
20 March 2020	#Corona, corona, coronavirus, #coronavirus
20 April 2020	#covid, covid, #covid19, covid 19, #covid-19, covid_19, #covid_19, 2019ncov, sars cov2, #2019-ncov

**Table 3:** Emojis shared via tweets.

Date	Tweet Tags	Unigram	Bigram	Trigram
20 March	deaths, people, covid positive	Index pointing	Folded hand	Backhand-index pointing
25 March	health, positive cases, curfew pandemic, tests, virus, masks,	Index-up-pointing	Folded hand	Backhand-index pointing
1 April	covid tests, stay home, impose curfew, fighting covid, health care, mask	Folded hand	Backhand-index pointing	Folded hand
5 April	brave, quarantine, be positive, mask, social distance	Index pointing	Folded hand	Backhand-index pointing
10 April	pandemic, testing, lockdown, positive, social distance, masks, death	Index-down-pointing	Folded hand	Folded hand
15 April	death rate increase, pandemic, coronavirus, people, government mask, social distance	Folded hand	Backhand-index pointing	Backhand-index pointing
20 April	brave, think positive, death, confidential, mask pandemic	Index pointing	Folded hand	Backhand-index pointing

**Table 4:** Performance of the unigram stacked autoencoder.

Ml Algorithm	Precision %	Recall %	F1-Score %
DT	81.1	81.12	81.48
SVM	83.48	81.76	80.74
RF	86.69	84.69	83.73
KNN	67.87	64.15	60.77

**Table 5:** Performance of bigram stacked auto encoder.

Ml Algorithm	Precision %	Recall %	F1 Score %
DT	81.91	82.25	81.48
SVM	85.08	81.76	80.95
RF	87.81	85.01	84.73
KNN	72.02	65.56	60.12

**Table 6:** Performance of trigram stacked autoencoder.

Ml Algorithm	Precision %	Recall %	F1-Score %
DT	82.91	82.56	81.98
SVM	84.34	83.76	81.93
RF	88.87	86.67	85.73
KNN	72.81	66.56	62.56

**Table 7:** Classification of threat level.

Threat Level	Sentiment Score (S)	Ratio of Negative Tweet
Low	S ≥ 0.2	r < 35%
Medium	−0.2 < S < 0.2	r ≥ 35%
High	S ≤ −0.2	r ≥ 45%

**Table 8:** Sample of detecting threats.

Tweet by People	Sentiment Score	Ratio of Negativity Tweets	Detecting Threats
User-A	−0.2466	0.66	Yes
User-B	−0.1989	0.5	Yes
User-C	−2564	0.17	No

## Data Availability

The historical tweets’ dataset was collected in duration from 20/03/2020 to 20/04/2020 from Twitter and filtered by #COVID-19 and #Coronavirus hashtags.

## References

[1] Zhang X., Saleh H., Younis E.M., Sahal R., Ali A.A. (2020). Predicting Coronavirus Pandemic in Real-Time Using Machine Learning and Big Data Streaming System. Hindawi Complex..

[2] Alamoodi A.H., Zaidan B.B., Zaidan A.A., Albahri O.S., Mohammed K.I., Malik R.Q., Almahdi E.M., Chyad M.A., Tareq Z., Albahri A.S. (2021). Sentiment analysis and its applications in fighting COVID-19 and infectious diseases: A systematic review. Expert Syst. Appl..

[3] Forbes 5G Networks and COVID-19 Coronavirus: Here Are the Latest Conspiracy Theories. https://www.forbes.com/sites/brucelee/2020/04/09/5g-networks-and-covid-19-coronavirus-here-are-the-latest-conspiracy-theories/?sh=47d7ce926d41.

[4] Brennen J.S., Simon F., Howard P.N., Nielsen R.K. (2020). Types, Sources, and Claims of COVID-19 Misinformation.

[5] Chawla S., Mittal M., Chawla M., Goyal L. (2020). Corona Virus-SARS-CoV-2: An Insight to Another way of Natural Disaster. EAI Endorsed Trans. Pervasive Health Technol..

[6] Mertens G., Gerritsen L., Duijndam S., Salemink E., Engelhard I.M. (2020). Fear of the coronavirus (COVID-19): Predictors in an online study conducted in March 2020. J. Anxiety Disord..

[7] Socio-Economic Impact of COVID-19|UNDP. https://www.undp.org/content/undp/en/home/coronavirus/socio-economic-impact-of-covid-19.html.

[8] Staszkiewicz P., Chomiak-Orsa I. (2020). Dynamics of the COVID-19 Contagion and Mortality: Country Factors, Social Media, and Market Response Evidence from a Global Panel Analysis. IEEE Access.

[9] Donthu N., Gustafsson A. (2020). Effects of COVID-19 on business and research. J. Bus. Res..

[10] Guo Y.-R., Cao Q.-D., Hong Z.-S., Tan Y.-Y., Chen S.-D., Jin H.-J., Tan K.-S., Wang D.-Y., Yan Y. (2020). The origin, transmission and clinical therapies on coronavirus disease 2019 (COVID-19) outbreak—An update on the status. Mil. Med. Res..

[11] Mittal M., Battineni G., Goyal L.M., Chhetri B., Oberoi S.V., Chintalapudi N., Amenta F. (2020). Cloud-based framework to mitigate the impact of COVID-19 on seafarers’ mental health. Int. Marit. Health.

[12] Akande O.N., Badmus T.A., Akindele A.T., Arulogun O.T. (2020). Dataset to support the adoption of social media and emerging technologies for students’ continuous engagement. Data Brief.

[13] Garcia L.P., Duarte E. (2020). Infodemic: Excess quantity to the detriment of quality of information about COVID-19. Epidemiol. Serv. Health.

[14] Hung M., Lauren E., Hon E.S., Birmingham W.C., Xu J., Su S., Hon S.D., Park J., Dang P., Lipsky M.S. (2020). Social Network Analysis of COVID-19 Sentiments: Application of Artificial Intelligence. J. Med. Internet Res..

[15] Mehmood R., See S., Katib I., Chlamtac I. (2020). Smart Infrastructure and Applications: Foundations for Smarter Cities and Societies.

[16] Shi Z., Rui H., Whinston A.B. (2014). Content Sharing in a Social Broadcasting Environment: Evidence from Twitter. MISQ.

[17] Boon-Itt S., Skunkan Y. (2020). Public Perception of the COVID-19 Pandemic on Twitter: Sentiment Analysis and Topic Modeling Study. JMIR Public Health Surveill.

[18] Plutchik R., Robert P., Henry K. (1980). A general psych evolutionary theory of emotion. Theories of Emotion.

[19] Lyu J., Han E., Luli G. (2021). COVID-19 Vaccine—Related Discussion on Twitter: Topic Modeling and Sentiment Analysis. J. Med. Internet Res..

[20] Jang H., Rempel E., Roth D., Carenini G., Janjua N. (2021). Tracking COVID-19 Discourse on Twitter in North America: Infodemiology Study Using Topic Modeling and Aspect-Based Sentiment Analysis. J. Med. Internet Res..

[21] Apuke O.D., Omar B. (2021). Fake news and COVID-19: Modelling the predictors of fake news sharing among social media users. Telemat. Inform..

[22] Zaman A. (2021). COVID-19-Related Social Media Fake News in India. J. Media.

[23] Depoux A., Martin S., Karafillakis E., Preet R., Wilder-Smith A., Larson H. (2020). The pandemic of social media panic travels faster than the COVID-19 outbreak. J. Travel Med..

[24] Gao J., Zheng P., Jia Y., Chen H., Mao Y., Chen S., Wang Y., Fu H., Dai J. (2020). Mental health problems and social media exposure during COVID-19 outbreak. PLoS ONE.

[25] Ahmad A.R., Murad H.R. (2020). The Impact of Social Media on Panic during the COVID-19 Pandemic in Iraqi Kurdistan: Online Questionnaire Study. J. Med. Internet Res..

[26] Cinelli M., Quattrociocchi W., Galeazzi A., Valensise C.M., Brugnoli E., Schmidt A.L., Zola P., Zollo F., Scala A. (2020). The COVID-19 social media infodemic. Sci. Rep..

[27] Twitter Twitter Usage Statistics—Internet Live Stats. https://www.internetlivestats.com/twitter-statistics/.

[28] Chakraborty K., Bhatia S., Bhattacharyya S., Platos J., Bag R., Hassanien A.E. (2020). Sentiment Analysis of COVID-19 tweets by Deep Learning Classifiers—A study to show how popularity is affecting accuracy in social media. Appl. Soft Comput..

[29] Shahsavari S., Holur P., Tangherlini T.R., Roychowdhury V. (2020). Conspiracy in the time of corona: Automatic detection of COVID-19 conspiracy theories in social media and the news. J. Comput. Soc. Sci..

[30] Havey N.F. (2020). Partisan public health: How does political ideology influence support for COVID-19 related misinformation?. J. Comput. Soc. Sci..

[31] Pinter G., Felde I., Mosavi A., Ghamisi P., Gloaguen R. (2020). COVID-19 pandemic prediction for Hungary; a hybrid machine learning approach. Mathematics.

[32] (2020). Twitter: Standard Search Api. https://developer.twitter.com/en/docs/tweets/search/overview.

[33] (2020). Twitter: Filter Real Time Tweets. https://developer.twitter.com/en/docs/tweets/filter-realtime/overview.

[34] Singh V., Kumar B., Patnaik T. (2013). Feature extraction techniques for handwritten text in various scripts: A survey. Int. J. Soft Comput. Eng..

[35] Trier D., Jain A.K., Taxt T. (1996). Feature extraction methods for character recognition—A survey. Pattern Recognit..

[36] Liang H., Sun X., Sun Y., Gao Y. (2017). Text feature extraction based on deep learning: A review. EURASIP J. Wirel. Commun. Netw..

[37] Kavinwidholm, Machine Learning Pipeline for Real-Time Sentiment Analysis. https://www.novatec-gmbh.de/en/blog/sentimentanalyzer/.

[38] Park W., You Y., Lee K. (2017). Twitter Sentiment Analysis Using Machine Learning, Research Briefs on Information & Communication Technology Evolution. http://rbisyou.wixsite.com/rebicte/volume-3-2017.

[39] Feng S., Kang J.S., Kuznetsova P., Choi Y. Connotation lexicon: A dash of sentiment beneath the surface meaning. Proceedings of the 51st Annual Meeting of the Association for Computational Linguistics.

[40] Losada M., Heaphy E. (2004). The Role of Positivity and Connectivity in the Performance of Business Teams: A Nonlinear Dynamics Model. Am. Behav. Sci..

[41] Park W., You Y., Lee K. (2018). Detecting Potential Insider Threat: Analyzing Insiders Sentiment Exposed in Social Media. Hindawi Secur. Commun. Netw..

[42] Venkatachalam K., Prabu P., Almutairi A., Abouhawwash M. (2021). Secure biometric authentication with de-duplication on distributed cloud storage. PeerJ Comput. Sci..

